# Prediction and Implications of Edoxaban-Associated Bleeding in Patients after Critical Illness

**DOI:** 10.3390/jcm12030860

**Published:** 2023-01-21

**Authors:** Ryusei Mikami, Mineji Hayakawa, Shungo Imai, Kunihiko Maekawa, Kojiro Yamazaki, Mitsuru Sugawara, Yoh Takekuma

**Affiliations:** 1 Department of Pharmacy, Hokkaido University Hospital, Sapporo 060-8648, Japan; 2 Department of Emergency Medicine, Hokkaido University Hospital, Sapporo 060-8648, Japan; 3 Faculty of Pharmacy, Keio University, Tokyo 105-8512, Japan; 4 Faculty of Pharmaceutical Sciences, Hokkaido University, Sapporo 060-0812, Japan

**Keywords:** critical illness, anticoagulant, edoxaban, bleeding score

## Abstract

In this retrospective study, we aimed to identify the risk factors for bleeding in patients after critical illness during edoxaban treatment. Data from patients who received edoxaban after critical illness at the Emergency Department at a tertiary care hospital were obtained from the hospital medical records. Multivariate analysis revealed the risk factors for edoxaban-associated bleeding. Additionally, we developed an edoxaban-associated bleeding score (EAB score) based on these results. The derived EAB score was compared with the HAS-BLED score using receiver operating characteristic (ROC) curve analysis. Bleeding was observed in 42 of 114 patients (36.8%). We identified the following bleeding predictors (odds ratios, 95% confidence interval, score points) using multivariate analysis: concomitant use of antiplatelet agents (6.759, 2.047–22.32, 2 points), concomitant use of P-glycoprotein inhibitors (3.825, 1.484–9.856, 1 point), prothrombin time (PT)% following edoxaban administration of <75% and ≥60% (2.507, 0.788–7.970, 1 point), and PT% following edoxaban administration of <60% (11.23, 3.560–35.42, 3 points). The ROC curve analysis revealed an area under the curve of 0.826 for the EAB score and 0.625 for the HAS-BLED score. Under appropriate edoxaban dosing regimens in patients after critical illness, a combination of antiplatelet agents, P-gp inhibitors, and a low PT% following edoxaban administration were identified as strong risk factors for bleeding.

## 1. Introduction

Direct oral anticoagulants (DOACs) are used to prevent stroke in patients with atrial fibrillation (AF) and to treat or prevent venous thromboembolism (VTE). DOACs are characterized by a lower incidence of bleeding complications compared with the traditionally used warfarin [1]. In addition, the influence of drug–drug interactions and food intake on the anticoagulant effect is smaller for DOACs than for warfarin, and frequent laboratory tests for dose adjustment are not necessary [2,3]. Despite these advantages, continuous assessment of coagulation ability during DOAC treatment is important to prevent bleeding complications [4,5]. Currently, the HAS-BLED score is widely used to predict major bleeding during anticoagulation [6]. However, it has been suggested that the HAS-BLED score is not sufficient to predict bleeding by DOACs [7].

Among DOACs, edoxaban is a relatively recently introduced drug, which is prescribed often [8,9,10]. Edoxaban has an oral bioavailability of 61.8%, with peak plasma concentrations 1 to 2 h after administration, an elimination half-life of 10–14 h, and 50% renal excretion [11,12,13]. Additionally, the anticoagulant effect of edoxaban is reflected, to some extent, in the prothrombin time (PT), although not as much as warfarin [14]. Some PT assays are less sensitive at trough plasma concentrations [15], but a decrease in PT% with no other explanation under edoxaban treatment indicates the presence of drug excess [16].

In Japan, the general dosage of edoxaban is 30–60 mg once daily. For patients with weight ≤ 60 kg, creatinine clearance (CrCl) of 15–50 mL/min, or concomitantly taking strong to moderate P-glycoprotein (P-gp) inhibitors, such as quinidine, verapamil, or amiodarone, a reduction in dosage to 15–30 mg once daily is recommended due to concerns about bleeding complications [17]. Nevertheless, even with the recommended dosing regimen, Asian patients are more prone to bleeding complications than subjects of other ethnicities [18,19,20]. Some studies suggested that Asian patients with low weight, cancer, and low baseline hemoglobin levels are at increased risk for edoxaban-associated bleeding [18,21,22], but the frequency of edoxaban-associated bleeding in emergency wards and the influence of patient background factors are unknown.

After critical conditions such as AF, trauma, surgery, or acute infection, many patients are prone to undergo thrombosis, and the need for anticoagulation therapy to prevent VTE in this population is high [23]. However, careful anticoagulation in these patients is usually required because of the presence of potential bleeding factors, including multiple organ damage, a recent history of bleeding, and concomitant use of agents that contribute to bleeding [24]. Thus, the purpose of this study was to identify risk factors for bleeding in patients after critical illness during edoxaban treatment.

## 2. Materials and Methods

### 2.1. Data Source

All data were obtained retrospectively from the medical records of patients who received edoxaban between April 2019 and June 2021 at the Emergency Department of Hokkaido University Hospital (a tertiary care hospital). Among clinically relatively stable inpatients after critical illness who had completed hyperacute treatment, edoxaban was administered to prevent stroke in patients with AF and treat or prevent VTE based on physicians’ judgments. The dosage of edoxaban was adjusted according to the indications provided in the package insert [17]. Patients were excluded under the following conditions: younger than 18 years, measurement deficiencies during the observation period, severe coagulofibrinolytic disorder at the start of edoxaban treatment, edoxaban treatment for less than 5 days in patients without bleeding, inappropriate dosing regimen. The maximum observation period before the occurrence of bleeding was 60 days.

### 2.2. Study Objective

The primary objective was to identify risk factors for bleeding in patients after critical illness during edoxaban treatment. Additionally, based on these results, we aimed to develop a simple predictive bleeding scoring model (hereafter called EAB score).

### 2.3. Bleeding Definition

The following three types of bleeding were defined: major bleeding, clinically relevant non-major bleeding, and minor bleeding. Major bleeding was defined according to the guidelines of the International Society on Thrombosis and Haemostasis (ISTH) as fatal bleeding, bleeding with a decrease in hemoglobin level of more than 2 g/dL, bleeding requiring transfusion of 2 or more units of blood, or bleeding at a critical site (intracranial, intrathoracic, retroperitoneal, or intraocular) [25]. Clinically relevant non-major bleeding was defined as bleeding not meeting the criteria for major bleeding that required physician care or resulted in a change in anticoagulants. Minor bleeding was defined as all bleeding not meeting criteria for major bleeding or clinically relevant non-major bleeding.

### 2.4. Measurement Method and Timing of PT

We used Quick’s PT method with Thromborel S reagent (Sysmex, Kobe, Japan) for PT measurements. PT was measured using sampling data collected 24 h after edoxaban administration.

### 2.5. Statistical Analysis

Continuous data are presented as median values and interquartile ranges (IQR). Categorical data are presented as counts (%). The Mann–Whitney U test was used to analyze the non-correspondence of continuous data. The Chi-squared test or Fisher’s exact test were used to analyze categorical data.

To identify the risk factors for edoxaban-associated bleeding, patient background and laboratory data were compared between patients with and without bleeding using univariate analysis. The variables selected by stepwise backward elimination were then subjected to multivariate analysis to identify the independent predictors of edoxaban-associated bleeding. Continuous variables selected as candidates for independent predictors were classified using a decision tree analysis to simplify the multivariate logistic regression analysis [26]. Multivariate analyses were performed using logistic regression to calculate odds ratios (OR) and 95% confidence intervals (CI) for risk factors associated with bleeding. Using the data obtained from the multivariate analysis, a weighted scoring system was developed. The performance of the developed model was evaluated in terms of discriminability and calibration. The discriminability of the model was compared with that of the HAS-BLED score using receiver operating characteristic (ROC) curve analysis, and sensitivity and specificity were calculated. The optimal cutoff value of the ROC curve was calculated using the Youden index [27]. Calibration was evaluated graphically, and the Hosmer–Lemeshow goodness-of-fit test was used to assess the fit of the model [28]. Internal validation of the multivariate model was performed using bootstrapping procedures with 1000 replicates [29]. Bootstrap samples were drawn with replacement from the original sample.

Two-sided *p*-values of less than 0.05 were considered statistically significant. Statistical analyses were performed using JMP version 15.0 statistical software (SAS Institute Inc., Cary, NC, USA) and R (version 4.0.2) software packages (R Foundation for Statistical Computing, Vienna, Austira).

## 3. Results

During the study period, 142 patients received edoxaban after critical illness. Of these, 28 were excluded for the following reasons: age < 18 years (*n* = 3), measurement deficiencies during the observation period (*n* = 9), severe coagulofibrinolytic disorder at the start of edoxaban treatment (*n* = 2), edoxaban treatment for less than 5 days in patients without bleeding (*n* = 7), and inappropriate dosing regimen (*n* = 7).

### 3.1. Patient Characteristics

Patient characteristics are shown in Table 1. In this study at a tertiary care hospital, patients with trauma were the most common (39%), followed by those with cardiovascular disease (22%), and central nervous system disease (11%). Bleeding was observed in 42 of 114 patients (36.8%). Of these, 10 (8.8%) had major bleeding, 25 (21.9%) had clinically relevant non-major bleeding, and 7 (6.1%) had minor bleeding. The median (IQR) occurrence of these bleeding events was 10 (7–20) days. The acute physiology and chronic health evaluation II (APACHE II) score [30] and CHA2DS2-VASc score [31] indicated that all patients were severely ill and required anticoagulation.

The HAS-BLED scores tended to be significantly higher in the bleeding group. There was a trend toward more concomitant use of strong to moderate P-gp inhibitors (verapamil, amiodarone) in the bleeding group, but the difference was not significant. In both groups, edoxaban doses were appropriately decreased based on the indications of the package insert in all patients under the concomitant use of strong to moderate P-gp inhibitors. The concomitant use of a weak P-gp inhibitor was significantly higher in the bleeding group. Suvorexant was the weak P-gp inhibitor used in all patients.

### 3.2. Laboratory Data

The patients’ laboratory data are shown in Table 2. The expression “following edoxaban administration” refers to the last laboratory data obtained before bleeding in the bleeding event group and after 5 days of edoxaban administration in the no event group. Edoxaban was administered once a day in the morning; measurements were performed at the trough phase of edoxaban prior to morning administration.

There were no significant differences between groups in the various coagulation parameters assessed at pre-dose edoxaban (Table 2). In the bleeding group, the prothrombin international normalized ratio (PT-INR) following edoxaban administration was significantly extended, and PT% following edoxaban administration was significantly lower. Likewise, blood urea nitrogen and serum creatinine were significantly higher in the bleeding group than in the non-bleeding group, and CrCl (Cockcroft–Gault equation [32]) was significantly lower.

### 3.3. Identifying Risk Factors for Edoxaban-Associated Bleeding

Those factors that showed a significant difference between patients with and without bleeding in the univariate analysis were subjected to backward stepwise elimination to identify candidates for independent predictors of edoxaban-associated bleeding. The concomitant use of antiplatelet agents and P-gp inhibitors and a low value for PT% following edoxaban administration were selected as candidates for independent predictors of edoxaban-associated bleeding events. Three PT% following edoxaban administration thresholds (≥75%, <75% and ≥60%, and <60%) were considered to identify risk factors for edoxaban-associated bleeding in patients after critical illness. Multivariate analysis confirmed that all these parameters were independent predictors (Table 3). The Hosmer–Lemeshow goodness-of-fit test was insignificant (*p* = 0.914), confirming the fit of the model.

### 3.4. Development of the EAB Score

**Figure 1:**
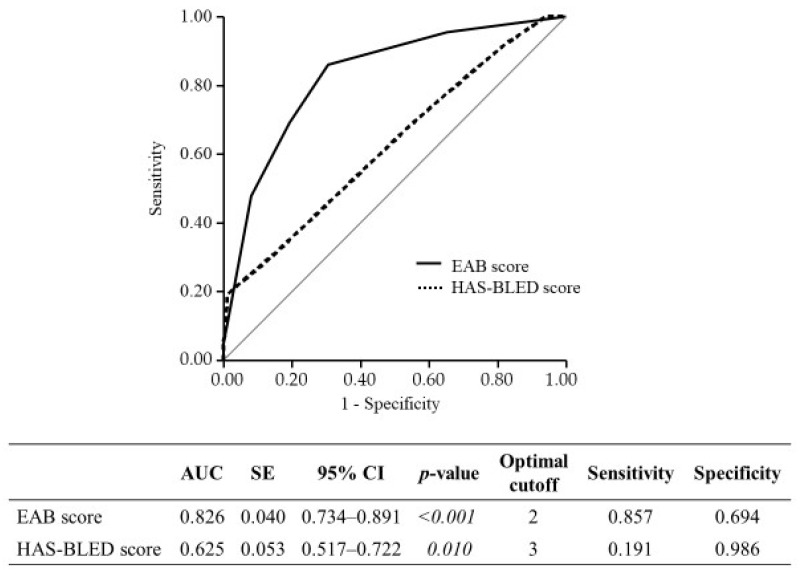
Receiver operating characteristic curves for EAB score and HAS-BLED score. Abbreviations: EAB, edoxaban-associated bleeding; AUC, area under the curve; SE, standard error; CI, confidence interval.

Based on the results of the multivariate analysis, we developed the following EAB score (indicated within parentheses): concomitant use of antiplatelet agents (2 points), concomitant use of P-glycoprotein inhibitors (1 point), PT% following edoxaban administration of <75% and ≥60% (1 point), and PT% following edoxaban administration <60% (3 points). The derived EAB score was a better predictive model for edoxaban-associated bleeding than the HAS-BLED score according to our area under the curve (AUC) data (Figure 1). Even when only major bleeding was included, the AUC (95% CI) of the EAB score was 0.667 (0.510–0.793), which was higher than the HAS-BLED score of 0.560 (0.378–0.727). Internal validation using the bootstrap procedure with 1000 data samples showed an optimized AUC of 0.806.

**Figure 2:**
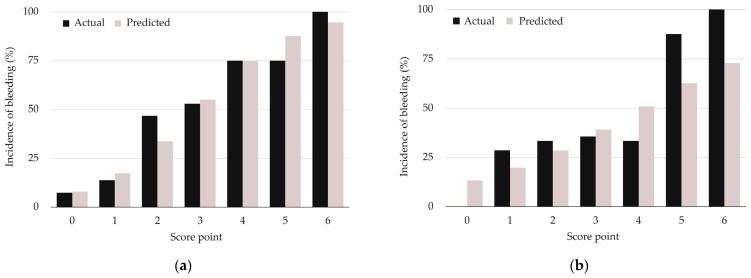
Predicted frequency of bleeding occurrence by (**a**) EAB score and (**b**) HAS-BLED score.

The predicted frequency and actual occurrence of bleeding at each point of the EAB and HAS-BLED scores are shown in Figure 2, respectively. In this study, none of the patients had a HAS-BLED score of 7 points or higher. It may be noticed that for HAS-BLED scores ≥ 4 points the predicted frequency of bleeding tended to diverge from the actual bleeding occurrence.

## 4. Discussion

This study aimed to identify the risk factors for edoxaban-associated bleeding in patients after critical illness during edoxaban treatment. As an additional analysis, we developed a scoring system to predict edoxaban-associated bleeding events in this population.

In this study involving patients after critical illness, concomitant use of antiplatelet agents, concomitant use of P-gp inhibitors, and a low value for PT% following edoxaban administration were identified as strong risk factors for edoxaban-associated bleeding. These risk factors were more strongly associated with bleeding than older age, hypertension, and decreased renal function. This may be due to the appropriate dose decrease in edoxaban according to the renal function in this study, which mitigated the effects of age and renal function. The Cockcroft–Gault formula used to evaluate renal function is greatly influenced by age. Additionally, this study included very few elderly (85 years or older) patients who were the subjects of the recent ELDERCARE-AF trial [33]. The degree of influence of known bleeding risk factors may vary under appropriate edoxaban dosing regimens in patients after critical illness.

In the present study, PT% during edoxaban therapy was significantly lower in the bleeding group. Edoxaban affects PT in a dose-dependent manner [16]. Edoxaban plasma concentration changes according to its pharmacokinetic profile, and PT values return to baseline levels during the trough phase [14]. Edoxaban-associated bleeding has been related to increased trough plasma concentrations [11,34,35]. Therefore, a decrease in PT% with no other explanation under edoxaban treatment is a risk factor for bleeding. It should be noted that these PT% following edoxaban administration values are laboratory data for the trough phase of edoxaban.

Bleeding risk is dynamic, and paying attention to changes in the bleeding risk profile is more important than simply relying on the baseline bleeding risk [36]. In a recent study, patients with a changed bleeding risk profile had a 3.5-fold higher risk of major bleeding in the first 3 months [37]. The usefulness of the dynamic bleeding-prediction score during edoxaban treatment described in this work is likely further enhanced in patients with acute conditions. Hence, PT% following edoxaban administration is an important dynamic index that may indicate an excessive effect during anticoagulation.

In the current study, the extent of concomitant use of weak P-gp inhibitors was significantly higher in patients with bleeding, and suvorexant was the weak P-gp inhibitor most frequently used. Previous reports and the package insert of edoxaban indicate that strong and moderate P-gp inhibitors can increase the plasma concentration of edoxaban [17,38]. Therefore, for patients concomitantly taking strong to moderate P-gp inhibitors, a reduction in the edoxaban dose is recommended. To the best of our knowledge, drug–drug interactions between edoxaban and suvorexant have not been reported to date. However, plasma concentration of digoxin (a P-gp substrate, as edoxaban) has been reported to increase 1.3-fold when combined with suvorexant [39]. Since the rate of increase in edoxaban and digoxin plasma concentrations was similar when these agents were used in combination with various P-gp inhibitors, a similar drug–drug interaction between edoxaban and suvorexant is likely to occur [38,40]. The contribution of P-gp to the oral clearance of edoxaban was reported to be as high as 50% [41]. Thus, even weak P-gp inhibitors may increase the risk of bleeding with edoxaban.

The newly developed EAB score can be easily determined at the bedside as the necessary factors can be easily determined at the bedside. Our intent was to include variables that are readily available and to avoid the use of past, uncertain information (e.g., medical history, prior major bleeding, alcohol use). The EAB score is simpler than the HAS-BLED score and can calculate the estimated risk of edoxaban-associated bleeding in patients after critical illness with sufficient discrimination and calibration.

In this study, the HAS-BLED score did not adequately reflect edoxaban-associated bleeding in patients after critical illness. In general, the HAS-BLED score (based on factors such as hypertension, abnormal renal/liver function, stroke, bleeding history or predisposition, labile PT-INR, being elderly, concomitant drug/alcohol use) is useful to some extent in estimating DOACs bleeding risk because many of these factors are risk factors for common anticoagulant therapy [6]. LaHaye et al. proposed an algorithm to stratify patients by combining the CHA2DS2-VASc score and the HAS-BLED score and selecting the optimal anticoagulation therapy based on the drug efficacy and price [42]. However, the HAS-BLED score is an assessment method for warfarin-treated patients and does not consider bleeding risk factors specific to DOACs. For example, it has been reported that patients with abnormal liver function may be at a higher risk of bleeding under warfarin treatment but possibly less so with DOACs [36]. Furthermore, it has been stated that labile PT-INR prior to anticoagulants is not a relevant factor for first-time DOAC users [7]. Claxton et al. reported that the predictive performance of HAS-BLED score in DOAC treatment is inadequate [7]. In line with that concept, we detected in this study that the AUC of the HAS-BLED score using ROC analysis was 0.625, which was not sufficient. On the other hand, the HAS-BLED score was developed to predict major bleeding, leaving aside clinically relevant non-major bleeding and minor bleeding, which constituted the most frequent bleeding events in this study. Even when only major bleeding was included, the predictive performance of the EAB score was better than that of the HAS-BLED score. Currently, there is no bleeding prediction score compatible with DOACs; nevertheless, the EAB score is an edoxaban-specific predictive scoring system that may be useful for patients with a high risk of bleeding after critical illness.

Currently, there is no bleeding score with sufficient predictive performance for anticoagulation with DOACs. This may also be due to differences in the pharmacokinetic profiles of DOACs. In this study, the decrease in PT% and concomitant use of P-gp inhibitors were selected as risk factors for edoxaban-associated bleeding. The contribution of P-gp to DOACs excretion is variable: P-gp has a relatively large contribution to edoxaban and dabigatran excretion (gastrointestinal-absorption inhibition and/or renal tubular secretion), whereas rivaroxaban and apixaban excretion (metabolism) are mainly dependent on cytochrome P450 [43]. DOACs also differ in terms of their influence on coagulation parameters. Edoxaban and rivaroxaban tend to affect PT%, whereas dabigatran tends to affect activated partial thromboplastin time (APTT) [15,16]. In this scenario, it is necessary to use different candidate bleeding factors according to the characteristics of individual DOACs or to monitor coagulation-function indicators that can be regularly assessed under all DOACs. Based on these considerations, the EAB score may be adequate for other target populations that receive edoxaban, but may not be appropriate for patients using other DOACs.

This study has some limitations. First, our results were not validated prospectively. Considering that they were conducted at a single center and in a limited population at a high risk of bleeding, the results should be interpreted with caution. The heterogeneity of the target patients was high, and the results from this small sample size may contain bias. Additionally, it should be noted that this study only included patients treated with edoxaban, which limits its generalizability to patients treated with other DOACs. Second, although edoxaban affects PT, as reported by Morishima et al. [44], there is variability among PT reagents. In general, anti-Xa activity is recommended for DOACs monitoring rather than PT. However, the routine measurement of anti-Xa activity in all patients is uncommon. Patients after critical illnesses are more likely to experience changes in coagulation activity associated with organ damage and impaired excretion of edoxaban. Moreover, although there is some variation among PT reagents, the decrease in PT% at trough phase after edoxaban administration suggests influence of the drug. In this sense, a trough PT% above a certain range would increase the potential for edoxaban accumulation. Considering the aforementioned reports and the results of this study, the EAB score may also be useful for screening anti-Xa activity. Specifically, the algorithm determines edoxaban overdose by measuring anti-Xa activity when the EAB score is high (positive PT% factor). We considered that the bleeding risk is hyperdynamic in patients after critical illness, and that paying attention to changes in the bleeding risk profile is important, for which a simple score is needed. Third, the risk-prediction-model development itself was limited by the small sample size and lack of external validation in this study. As with any predictive modeling study, it is important to assess the characteristics of the target patient population. Our study population consisted of patients after critical illnesses in tertiary care hospitals. Despite the clinical importance of this predictive score, the heterogeneity of these patients was high and rare. Therefore, the new EAB score needs to be validated externally by accumulating sufficient cases and should only be utilized in similar populations until validated in other cohorts. Fourth, no reports on drug interactions between edoxaban and suvorexant have been published up to now. We plan to verify this through in vitro or in vivo future studies. Finally, no ischemic events were observed in this study, so the efficacy of edoxaban to prevent such events could not be fully assessed. In general, the risk factors for thrombosis and bleeding are similar. For that reason, it is important to carefully balance each patient’s risk of thrombosis and bleeding when administering anticoagulation.

## 5. Conclusions

The EAB score is a simple and practical scoring system for predicting edoxaban-associated bleeding in patients after critical illness.

## Figures and Tables

**Table 1:** Patient characteristics.

	All	Bleeding Events	No Events	*p*-Value
Age, years ^a^	69 (48–78)	72 (63–81)	66 (40–77)	*0.031*
Female gender ^b^	48 (42)	18 (43)	30 (42)	*1.000*
Height, cm ^a^	162 (153–167)	159 (152–165)	163 (153–168)	*0.207*
Weight, kg ^a^	57 (48–69)	55 (48–64)	59 (49–70)	*0.159*
BSA, m^2 a^	1.60 (1.44–1.75)	1.55 (1.41–1.68)	1.64 (1.46–1.76)	*0.119*
BMI, kg/m^2 a^	22 (20–25)	23 (20–25)	22 (20–26)	*0.718*
APACHE II score ^a^	28 (19–31)	30 (21–32)	28 (19–31)	*0.475*
ISTH DIC score ^a^	3 (2–4)	3 (2–4)	3 (2–4)	*0.277*
CHA_2_DS_2_-VASc score ^a^	7 (6–9)	7 (6–9)	7 (6–8)	*0.983*
HAS-BLED score ^a^	3 (2–3)	3 (2–4)	3 (2–3)	*0.028*
Edoxaban daily dose, mg ^b^				
60	22 (19)	8 (19)	14 (19)	*0.912*
30	83 (73)	30 (71)	53 (74)	*0.656*
15	9 (7.9)	4 (9.5)	5 (6.9)	*0.649*
Admission diagnosis ^b^				
Cardiovascular diseases	25 (22)	11 (26)	14 (19)	*0.483*
Respiratory failure	8 (7.0)	1 (2.4)	7 (9.7)	*0.255*
Central nervous system diseases	12 (11)	4 (9.5)	8 (11)	*1.000*
Digestive diseases	5 (4.4)	2 (4.8)	3 (4.2)	*1.000*
Trauma	45 (39)	12 (29)	33 (46)	*0.077*
Infection	11 (9.6)	7 (17)	4 (5.6)	*0.096*
Other	8 (7.0)	5 (12)	3 (4.2)	*0.145*
Medical history ^b^				
Heart and/or respiratory failure	32 (28)	13 (31)	19 (26)	*0.668*
Hypertension	63 (55)	33 (79)	30 (42)	*<0.001*
Diabetes	34 (30)	17 (40)	17 (24)	*0.089*
Previous ischemic stroke or TIA	21 (18)	11 (26)	10 (14)	*0.134*
Already known thrombophilic condition	25 (22)	12 (29)	13 (18)	*0.242*
Acute infection and/or rheumatologic dis-order	71 (62)	28 (67)	43 (60)	*0.549*
Reduced mobility	113 (99)	41 (98)	72 (100)	*0.368*
Recent (≤1 month) trauma and/or surgery	95 (83)	35 (83)	60 (83)	*1.000*
Previous bleeding	80 (70)	32 (76)	48 (67)	*0.396*
Cancer	17 (15)	10 (24)	7 (9.7)	*0.057*
Medication ^b^				
Antiplatelet agents	21 (18)	14 (33)	7 (9.7)	*0.003*
NSAIDs	23 (20)	7 (17)	16 (22)	*0.630*
Steroids	5 (4.4)	1 (2.4)	4 (5.6)	*0.650*
PPI or H2RA	95 (83)	37 (88)	58 (81)	*0.435*
P-gp inhibitor	50 (44)	27 (64)	23 (32)	*0.003*
Strong to moderate P-gp inhibitor	6 (5.3)	3 (7.1)	3 (4.2)	*0.668*
Weak P-gp inhibitor	44 (39)	24 (57)	20 (28)	*0.003*

^a^ Median (interquartile range), ^b^ number (%). Abbreviations: BSA, body surface area; BMI, body mass index; APACHE II, acute physiology and chronic health evaluation II; ISTH, International Society on Thrombosis and Haemostasis; TIA, transient ischemic attack; NSAIDs, non-steroidal anti-inflammatory drugs; P-gp, P-glycoprotein; PPI, proton pump inhibitor; H2RA, histamine H2 receptor antagonist.

**Table 2:** Laboratory data.

	All	Bleeding Events	No Events	*p*-Value
White blood cell, ×10^3^/μL	9.5 (7.2–11.6)	8.7 (6.8–10.8)	9.6 (7.2–12.0)	*0.264*
Hemoglobin, g/dL	9.8 (9.0–11.1)	9.7 (8.7–10.4)	9.8 (9.0–11.4)	*0.236*
Platelet, ×10^3^/μL	234 (141–378)	223 (138–361)	236 (155–403)	*0.436*
Fibrinogen, mg/dL	481 (396–570)	465 (369–539)	493 (406–609)	*0.317*
Antithrombin, %	89 (79–104)	89 (77–103)	91 (81–111)	*0.447*
Fibrin/fibrinogen degradation products, μg/mL	13.9 (8.1–22.1)	12.1 (7.2–19.6)	15.3 (8.6–26.5)	*0.159*
D-dimer, μg/mL	7.1 (4.3–13.5)	5.86 (3.6–10.4)	8.8 (5.3–14.9)	*0.064*
PT%	88 (78–98)	85 (75–95)	89 (80–101)	*0.051*
PT-INR	1.07 (1.01–1.14)	1.10 (1.02–1.17)	1.06 (0.99–1.13)	*0.081*
APTT, sec	30 (28–33)	31 (28–34)	30 (28–33)	*0.286*
PT% following edoxaban administration	71 (58–85)	59 (48–69)	78 (65–88)	*<0.001*
PT-INR following edoxaban administration	1.21 (1.09–1.36)	1.37 (1.23–1.47)	1.15 (1.07–1.26)	*<0.001*
APTT following edoxaban administration, sec	32 (30–35)	33 (30–36)	32 (30–34)	*0.070*
Bilirubin, mg/dL	0.7 (0.5–1.0)	0.7 (0.5–1.0)	0.6 (0.5–1.1)	*0.795*
Aspartate aminotransferase, U/L	44 (27–89)	40 (26–62)	48 (28–102)	*0.215*
Alanine aminotransferase, U/L	38 (22–67)	32 (19–60)	40 (25–69)	*0.165*
Blood urea nitrogen, mg/dL	21 (15–32)	28 (18–38)	18 (13–30)	*0.002*
Serum creatinine concentration, mg/dL	0.70 (0.55–0.96)	0.86 (0.62–1.17)	0.64 (0.50–0.90)	*0.004*
Creatinine clearance, mL/min	78 (51–120)	56.7 (39.6–83.2)	94.4 (54.8–120)	*<0.001*

All values indicate median (interquartile range). Abbreviations: PT, prothrombin time; INR, international normalized ratio; APTT, activated partial thromboplastin time.

**Table 3:** Multivariate analyses of factors associated with bleeding.

	Multivariate Analysis		
	Odds Ratio (95% Confidence Interval)	Beta	*p*-Value
Antiplatelet agents	6.759 (2.047–22.32)	1.9	*0.002*
P-gp inhibitors	3.825 (1.484–9.856)	1.3	*0.006*
PT% following edoxaban administration			
≥75%	Reference	-	-
≥60% and <75%	2.507 (0.788–7.970)	0.9	*0.120*
<60%	11.23 (3.560–35.42)	2.4	*<0.001*

Abbreviations: PT, prothrombin time; INR, international normalized ratio; APTT, activated partial thromboplastin time.

## Data Availability

The datasets used and/or analyzed during the current study are available from the corresponding author on reasonable request.

## References

[1] January C.T., Wann L.S., Alpert J.S., Calkins H., Cigarroa J.E., Cleveland J.C., Conti J.B., Ellinor P.T., Ezekowitz M.D., Field M.E. (2014). 2014 AHA/ACC/HRS guideline for the management of patients with atrial fibrillation: A report of the American College of Cardiology/American Heart Association Task Force on practice guidelines and the Heart Rhythm Society. Circulation.

[2] Drouet L., Bal dit Sollier C., Steiner T., Purrucker J. (2016). Measuring non-vitamin K antagonist oral anticoagulant levels: When is it appropriate and which methods should be used?. Int. J. Stroke.

[3] Eikelboom J.W., Quinlan D.J., Hirsh J., Connolly S.J., Weitz J.I. (2017). Laboratory Monitoring of Non-Vitamin K Antagonist Oral Anticoagulant Use in Patients with Atrial Fibrillation: A Review. JAMA Cardiol..

[4] Chen A., Stecker E., Warden A.B. (2020). Direct Oral Anticoagulant Use: A Practical Guide to Common Clinical Challenges. J. Am. Heart Assoc..

[5] Corsini A., Ferri N., Proietti M., Boriani G. (2020). Edoxaban and the Issue of Drug-Drug Interactions: From Pharmacology to Clinical Practice. Drugs.

[6] Pisters R., Lane D.A., Nieuwlaat R., de Vos C.B., Crijns H.J., Lip G.Y. (2010). A novel user-friendly score (HAS-BLED) to assess 1-year risk of major bleeding in patients with atrial fibrillation: The Euro Heart Survey. Chest.

[7] Claxton J.S., MacLehose R.F., Lutsey P.L., Norby F.L., Chen L.Y., O’Neal W.T., Chamberlain A.M., Bengtson L.G.S., Alonso A. (2018). A new model to predict major bleeding in patients with atrial fibrillation using warfarin or direct oral anticoagulants. PLoS ONE.

[8] Ruff C.T., Giugliano R.P., Antman E.M., Crugnale S.E., Bocanegra T., Mercuri M., Hanyok J., Patel I., Shi M., Salazar D. (2010). Evaluation of the novel factor Xa inhibitor edoxaban compared with warfarin in patients with atrial fibrillation: Design and rationale for the Effective aNticoaGulation with factor xA next GEneration in Atrial Fibrillation-Thrombolysis in Myocardial Infarction study 48 (ENGAGE AF-TIMI 48). Am. Heart J..

[9] Giugliano R.P., Ruff C.T., Braunwald E., Murphy S.A., Wiviott S.D., Halperin J.L., Waldo A.L., Ezekowitz M.D., Weitz J.I., Špinar J. (2013). Edoxaban versus warfarin in patients with atrial fibrillation. N. Engl. J. Med..

[10] Ruff C.T., Giugliano R.P., Braunwald E., Morrow D.A., Murphy S.A., Kuder J.F., Deenadayalu N., Jarolim P., Betcher J., Shi M. (2015). Association between edoxaban dose, concentration, anti-Factor Xa activity, and outcomes: An analysis of data from the randomised, double-blind ENGAGE AF-TIMI 48 trial. Lancet.

[11] Yin O.Q., Tetsuya K., Miller R. (2014). Edoxaban population pharmacokinetics and exposure-response analysis in patients with non-valvular atrial fibrillation. Eur. J. Clin. Pharmacol..

[12] Matsushima N., Lee F., Sato T., Weiss D., Mendell J. (2013). Bioavailability and Safety of the Factor Xa Inhibitor Edoxaban and the Effects of Quinidine in Healthy Subjects. Clin. Pharmacol. Drug Dev..

[13] Bathala M.S., Masumoto H., Oguma T., He L., Lowrie C., Mendell J. (2012). Pharmacokinetics, biotransformation, and mass balance of edoxaban, a selective, direct factor Xa inhibitor, in humans. Drug Metab. Dispos..

[14] Ogata K., Mendell-Harary J., Tachibana M., Masumoto H., Oguma T., Kojima M., Kunitada S. (2010). Clinical safety, tolerability, pharmacokinetics, and pharmacodynamics of the novel factor Xa inhibitor edoxaban in healthy volunteers. J. Clin. Pharmacol..

[15] Samuelson B.T., Cuker A., Siegal D.M., Crowther M., Garcia D.A. (2017). Laboratory Assessment of the Anticoagulant Activity of Direct Oral Anticoagulants: A Systematic Review. Chest.

[16] Douxfils J., Ageno W., Samama C.M., Lessire S., Cate H.T., Verhamme P., Dogné J.M., Mullier F. (2018). Laboratory testing in patients treated with direct oral anticoagulants: A practical guide for clinicians. J. Thromb. Haemost..

[17] Pharmaceuticals and Medical Devices Agency (2021). LIXIANA® [Package Insert] (Daiichi-Sankyo, Inc., Tokyo, Japan). https://www.info.pmda.go.jp/go/pack/3339002F4029_1_08.

[18] Lee O.S., Kim W., Jang B.M., Min K.H., Cho Y.S., Lee M.K., Lee K.E. (2021). Association of risk factors and bleeding complications in Asian patients taking edoxaban. Br. J. Clin. Pharmacol..

[19] Bang O.Y., Hong K.S., Heo J.H., Koo J., Kwon S.U., Yu K.H., Bae H.J., Lee B.C., Yoon B.W., Kim J.S. (2014). New oral anticoagulants may be particularly useful for asian stroke patients. J. Stroke.

[20] Ayala C., Croft J.B., Greenlund K.J., Keenan N.L., Donehoo R.S., Malarcher A.M., Mensah G.A. (2002). Sex differences in US mortality rates for stroke and stroke subtypes by race/ethnicity and age, 1995–1998. Stroke.

[21] Yamashita T., Koretsune Y., Yasaka M., Inoue H., Kawai Y., Yamaguchi T., Uchiyama S., Matsumoto M., Ogawa S. (2012). Randomized, multicenter, warfarin-controlled phase II study of edoxaban in Japanese patients with non-valvular atrial fibrillation. Circ. J..

[22] Takase T., Ikesue H., Nakagawa H., Kinoshita M., Muroi N., Kitai T., Furukawa Y., Hashida T. (2020). Risk Factors for Major Bleeding and Clinically Relevant Non-major Bleeding in Japanese Patients Treated with Edoxaban. Biol. Pharm. Bull..

[23] Geerts W.H., Bergqvist D., Pineo G.F., Heit J.A., Samama C.M., Lassen M.R., Colwell C.W. (2008). Prevention of venous thromboembolism: American College of Chest Physicians Evidence-Based Clinical Practice Guidelines (8th Edition). Chest.

[24] Decousus H., Tapson V.F., Bergmann J.F., Chong B.H., Froehlich J.B., Kakkar A.K., Merli G.J., Monreal M., Nakamura M., Pavanello R. (2011). Factors at admission associated with bleeding risk in medical patients: Findings from the IMPROVE investigators. Chest.

[25] Schulman S., Kearon C. (2005). Definition of major bleeding in clinical investigations of antihemostatic medicinal products in non-surgical patients. J. Thromb. Haemost..

[26] Song Y.Y., Lu Y. (2015). Decision tree methods: Applications for classification and prediction. Shanghai Arch. Psychiatry..

[27] Youden W.J. (1950). Index for rating diagnostic tests. Cancer.

[28] Hosmer D.W., Hosmer T., Cessie L.S., Lemeshow S. (1997). A comparison of goodness-of-fit tests for the logistic regression model. Stat. Med..

[29] Harrell F.E., Lee K.L., Mark D.B. (1996). Multivariable prognostic models: Issues in developing models, evaluating assumptions and adequacy, and measuring and reducing errors. Stat. Med..

[30] Knaus W.A., Draper E.A., Wagner D.P., Zimmerman J.E. (1985). APACHE II: A severity of disease classification system. Crit. Care. Med..

[31] Camm A.J., Kirchhof P., Lip G.Y., Schotten U., Savelieva I., Ernst S., Van Gelder I.C., Al-Attar N., Hindricks G., Prendergast B. (2010). Guidelines for the management of atrial fibrillation: The Task Force for the Management of Atrial Fibrillation of the European Society of Cardiology (ESC). Eur. Heart J..

[32] Cockcroft D.W., Gault M.H. (1976). Prediction of creatinine clearance from serum creatinine. Nephron.

[33] Okumura K., Akao M., Yoshida T., Kawata M., Okazaki O., Akashi S., Eshima K., Tanizawa K., Fukuzawa M., Hayashi T. (2020). ELDERCARE-AF Committees and Investigators. Low-Dose Edoxaban in Very Elderly Patients with Atrial Fibrillation. N. Engl. J. Med..

[34] Salazar D.E., Mendell J., Kastrissios H., Green M., Carrothers T.J., Song S., Patel I., Bocanegra T.S., Antman E.M., Giugliano R.P. (2012). Modelling and simulation of edoxaban exposure and response relationships in patients with atrial fibrillation. Thromb. Haemost..

[35] Weitz J.I., Connolly S.J., Patel I., Salazar D., Rohatagi S., Mendell J., Kastrissios H., Jin J., Kunitada S. (2010). Randomised, parallel-group, multicentre, multinational phase 2 study comparing edoxaban, an oral factor Xa inhibitor, with warfarin for stroke prevention in patients with atrial fibrillation. Thromb. Haemost..

[36] Hindricks G., Potpara T., Dagres N., Arbelo E., Bax J.J., Blomström-Lundqvist C., Boriani G., Castella M., Dan G.A., Dilaveris P.E. (2021). 2020 ESC Guidelines for the diagnosis and management of atrial fibrillation developed in collaboration with the European Association for Cardio-Thoracic Surgery (EACTS): The Task Force for the diagnosis and management of atrial fibrillation of the European Society of Cardiology (ESC) Developed with the special contribution of the European Heart Rhythm Association (EHRA) of the ESC. Eur. Heart J..

[37] Chao T.F., Lip G.Y.H., Lin Y.J., Chang S.L., Lo L.W., Hu Y.F., Tuan T.C., Liao J.N., Chung F.P., Chen T.J. (2018). Incident Risk Factors and Major Bleeding in Patients with Atrial Fibrillation Treated with Oral Anticoagulants: A Comparison of Baseline, Follow-up and Delta HAS-BLED Scores with an Approach Focused on Modifiable Bleeding Risk Factors. Thromb. Haemost..

[38] Mendell J., Zahir H., Matsushima N., Noveck R., Lee F., Chen S., Zhang G., Shi M. (2013). Drug-drug interaction studies of cardiovascular drugs involving P-glycoprotein, an efflux transporter, on the pharmacokinetics of edoxaban, an oral factor Xa inhibitor. Am. J. Cardiovasc. Drugs.

[39] Pharmaceuticals and Medical Devices Agency (2021). Belsomra® [Package Insert] (MSD, Inc., Tokyo, Japan). https://www.info.pmda.go.jp/go/pack/1190023F1024_1_14.

[40] Tanaka H., Matsumoto K., Ueno K., Kodama M., Yoneda K., Katayama Y., Miyatake K. (2003). Effect of clarithromycin on steady-state digoxin concentrations. Ann. Pharmacother..

[41] Mikkaichi T., Yoshigae Y., Masumoto H., Imaoka T., Rozehnal V., Fischer T., Okudaira N., Izumi T. (2014). Edoxaban transport via P-glycoprotein is a key factor for the drug’s disposition. Drug Metab. Dispos..

[42] LaHaye S.A., Gibbens S.L., Ball D.G., Day A.G., Olesen J.B., Skanes A.C. (2012). A clinical decision aid for the selection of antithrombotic therapy for the prevention of stroke due to atrial fibrillation. Eur. Heart J..

[43] Terrier J., Gaspar F., Fontana P., Youssef D., Reny J.L., Csajka C., Samer C.F. (2021). Drug-Drug Interactions with Direct Oral Anticoagulants: Practical Recommendations for Clinicians. Am. J. Med..

[44] Morishima Y., Kamisato C. (2015). Laboratory measurements of the oral direct factor Xa inhibitor edoxaban: Comparison of prothrombin time, activated partial thromboplastin time, and thrombin generation assay. Am. J. Clin. Pathol..

